# Determinants of postoperative emergence delirium in patient undergoing general anesthesia in Dilla University referral hospital. A case-control study

**DOI:** 10.1016/j.amsu.2022.104942

**Published:** 2022-11-14

**Authors:** Teshome Regasa, Zemedu Aweke, Derartu Neme

**Affiliations:** Dilla University, College of Medicine and Health Science, Dilla, Ethiopia

**Keywords:** Postoperative delirium, Delirium risk factor, Elderly people and delirium

## Abstract

**Background:**

Delirium is affecting of concentration, decreases the ability to forward-thinking, attention, sustainability, change, and decreases orientation to the environment. Delirium has a serious impact on the overall outcome of the patient. Post-operative emergence delirium (POED) increases hospital mortality by 5% and post-discharge hospitalization by 33%, compared to those without postoperative delirium. Postoperative delirium incidence has different summative risk factors and recognizing the multiple risks of delirium complications may help the clinician to design supportive measures to prevent delirium. Delirium can cause a series of outcomes and is increase the length of hospital stay, independent predictor for intensive care unit (ICU) admission and institutional morbidity and mortality, increase institutional care, for those patients and increase hospital expenses.

**Methodology:**

Unmatched case-control study was employed from September 2019 to October 2020. This study was conducted on 264 patients above 18years. A structured questionnaire prepared in English was used for data collection. Data were analyzed by using the SPSS software. Bivariate and multiple logistic regression models were used to identify associated risk factors for incidence of POED and a P-Value of less than 0.05 was the risk factor for this medical condition.

**Result:**

Out of 264 participants included in the study 56.4% were female. ASA I and II constitute 97.4%. Substance abuse, premediate with diazepam, & ketamine were high risk for POD with p-value of 0.000, 0.005, & 0.047 respectively.

**Conclusion:**

We conclude that older age, current substance use, Coexisting disease, Benzodiazepine exposures, Ketamine, ASA physical status, and coexisting disease were determinant risk factors for postoperative delirium clients undergoing general anesthesia.

## Highlights

• Substance abuse was a high risk for postoperative emergence delirium.
• Premedicated with diazepam was high risk for postoperative emergence delirium.
• Induction with ketamine was a high risk for postoperative emergence delirium.

## 1 Background

Delirium is affecting of concentration, decreases the ability to forward-thinking, attention, sustainability, change, and decrease orientation to the environment [1]. Delirium has been subdivided into hyperactive delirium which was characterized by agitation and restlessness. The other is hypoactive delirium in which lethargy was the main future. The mixed type consists of both futures [2]. General anesthesia (GA) is a state of hypnosis, free of pain, and sensation, throughout the whole body by the delivery of anesthetic drugs for some medical conditions and surgical activities [3]. The incidence of postoperative delirium varies from 0 to 73.5%. The incidence varies with the varying degree of predictors. Perioperative factor associated with POED is classified as preoperative, intraoperative, and postoperative factors [4]. Although the mechanism is not known, different literature described several patients' associated risk factors. Noxious injury, previous history of POED, AGE >70, preexisting cognitive impairments, preoperative use of narcotics, benzodiazepines, alcohol use, glucose and electrolyte disturbance, GA, hemodynamic instability, high-stress procedure (major operation), pain, infections In 40% of the cases postoperative delirium is preventable [5].

Delirium has a serious impact on the overall outcome of the patient. POED increases hospital mortality by 5% and post-discharge hospitalization by 33%, compared to those without POED. POED prolongs the mean ICU stay time by 5.3 days and increases the length of hospitalization by 8.4 days compared to those without POED [6]. The emergence of delirium can result in inadvertent removal of an intravenous catheter, drains, dressing, and might cause self-harm [7]. 40% of clients develop delirium during the preoperative, intraoperative, and postoperative time [8]. Previously some predictors of POED were studied & different ways of management of POED were considered, starting from prevention to proper management with different strategies like risk assessment, stratification, giving care for risk patients on the ward and, prophylactic appropriate before surgical procedure. From an anesthetic perspective, evidence in some surgical populations would support the use of regional techniques and minimal sedation. If delirium develops, treatment with low-dose oral antipsychotics appears to be most effective in the developed country [9]. Different risk factors for the incidence of delirium may help the clinician to design supportive measures to prevent delirium and managements of POED.

## 2 Objectives

### 2.1 General objective

Determine predictors of postoperative emergence delirium in a patient undergoing general anesthesia in Dilla university referral hospital from September 2019 to October 2020.

### 2.2 Specific objective

To determine predictors of postoperative emergence delirium.

## 3 Method and materials

### 3.1 Design and clients

An unmatched case-control study was done from August 2019 to October 2020 in xxxx hospital. xxxxx hospital institutional review board obtained ethical clearance. Informed consent was taken from each client. All surgical patients scheduled for the surgical procedure with general anesthesia were participate in this study. The seven-month consecutive report in xxxx hoapital showed 700 patients undergoing general anesthesia surgery. All patients have a 33% equal chance to involve in the study since the sample size was 264. By using systematic random sampling the k value was (k = N/n, 700/264 = 2.7 ≈ 3), where N = number of patients during the study period, n = sample size, k = interval. Of the three patients, one patient was selected through a lottery method to the study participant. Spinal anesthesia that was converted to general anesthesia because of total spinal or inadequate block and Age less than 18 years were excluded from the study. Socio-demographic data like the client's age, sex, and ASA status, type of anesthesia and surgical procedure, duration of anesthesia and surgery, associated coexisting illness were recorded from the chart. Data was collected from Post Anesthesia Care Unit. Using systematic sampling techniques by using admission sequence the data collection procedure included chart review, and RASS measurement, which was used to perform the degree of agitation and sedation in ICU clients, to evaluate delirium in the PACU using a structured questionnaire prepared in English. Patients were classified as a **case** when the RASS score of -1-5 (hypoactive delirium) and score 1–4 (hyperactive delirium) and **control**led when the score is 0. The patients were followed immediately after extubation and throughout PACU stay and post-operative delirium was diagnosed based on RASS score. STROCSS 2021 checklist was used to state our completed activities by each item [10] and the manuscript registration number was 7363 https://www.researchregistry.com/browse-the-registry#home/.

### 3.2 Variables

**Independent variables**.

- Age, BMI, Pregnancy,
- ASA status, indication for surgery
- Baseline vital signs, intraoperative hypotension
- Premedication
- Surgery time
- Anesthesia drugs
- Perioperative analgesia
- Procedural urgency

**Dependent variables**.

- Postoperative emergence Delirium

### 3.3 Sample size determination

The sample size was determined with Epi-info analysis from a previous study of a different predictor of POED using preoperative benzodiazepine. Two-side confidence level 95% with the power of 80%, the ratio of control to case 1:2, percent of control exposed 6.7%, percent of the case with exposure 21.4% with OR of 3.79. So from this calculation sample size is 176 patients for control & 88 patients for cases & a total sample size of 264 patients [11].

### 3.4 Data analysis

Statistical analyses were performed using the statistical package SPSS. Continuous variables were expressed as mean ± standard deviation and categorical variables were presented as percentages. A multivariate logistic regression model was used to identify the most important risk factors for developing POED including variables with a significant trend on bivariate analysis and a P-Value of less than 0.05 was considered as significantly associated with the outcome variable.

## 4 Result

**Fig. 1:**
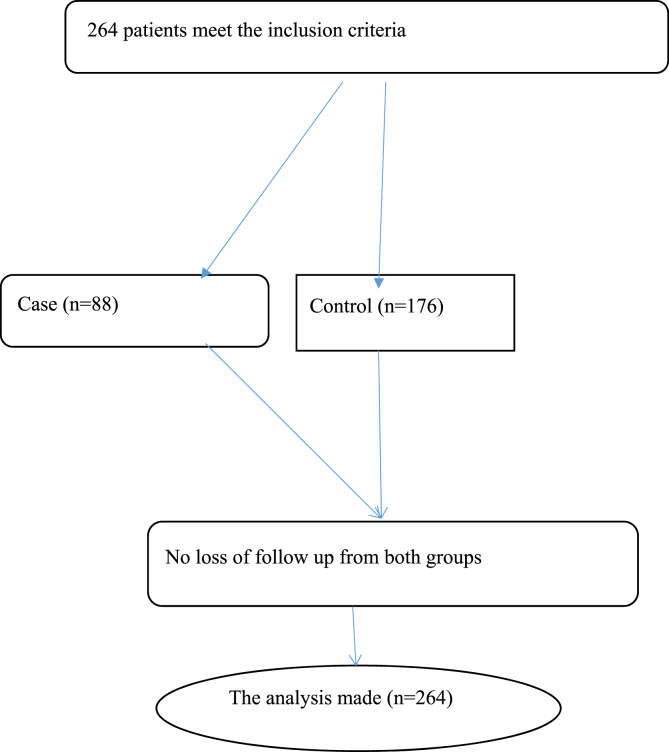
Consort diagram for patients selection.

The following figure is showing the patient's selection criteria for inclusion and exclusion (Fig. 1).

Out of 264 participants included in the study, 56.4% were female. ASA I and II constitute 97.4% of patients as shown in Table 1.

**Table 1:** Sociodemographic characteristics of patients operated on March 6 in xxxx hospital to May 12, 2020.

Variables	Groups	Frequency (%)
sex	Male	115(43.6)
	Female	149(56.4)
Age group	18–40	113 (42.8)
	41–64	121(45.8)
	>64	30 (11.4)
ASA	ASA I	159 (60.2)
	ASA II	100 (37.9)
	ASA III	5(1.9)
Coexisting disease	Yes	67(25.4)
	No	197 (74.6)
Social history of substance abuse	Yes	79 (29.9)
	No	185 (70.1)
Preoperative anxiety	Yes	52(19.7)
	No	212(80.3)

### 4.1 Anesthesia and surgery-related characteristics

Thiopental and Propofol constitute 78.4% of induction drugs used while the rest were induced with ketamine and ketofol agent with percentages of 5.7% and 15.9% respectively. The median (IQR) for the duration of anesthesia is 120 (100) minutes while the median and IQR for the duration of surgery were 90 and 90 min respectively. GA with ETT was among the commonest type of anesthesia employed as in Table 2.

**Table 2:** Surgical and anesthesia procedure-related variables in study participants.

Variables		Frequency (%)
Types of surgery	General surgery	110(41)
	Gynecology	66 (25)
	Neurosurgery	8(3)
	Orthopedics	53 (20.1)
	Urology	24 (9.1)
Types of anesthesia	GA with ETT	234 (88.4)
	GA with LMA	9 (3.4)
	Inhalational	3 (1.1)
	MAC	5 (1.9)
	TIVA	13(4.9)
Postoperative analgesia uses	Yes	190 (72)
	No	74 (28)
Presence of tracheal tube	Yes	5 (1.9)
	No	259(98.1)
Presence of urinary catheters	Yes	186 (69.3)
	No	81 (30.7)
Premedication with benzodiazepines	Yes	25 (9.5)
	No	239(90.5)

The Socio-demographic and preoperative risks of Postoperative delirium by using Crude Odds Ratio (COR). All variables with p-values less than 0.2 were recruited for multivariate analysis and presented using Adjusted Odds Ratio (AOR) along with 95% confidence intervals and P-values of the adjusted Odds ratio of substance abuse, premedication with diazepam, and age >65 years were 0.000, 0.005, and 0.010 respectively as shown Table 3.

**Table 3:** Socio-demographic and preoperative risks of Postoperative.

variable		COR (95% CI)	P-value	AOR (95% CI)	P-value
Age groups	**18**–**40 #**	1			
	40–64	2.485 (1.383–4.465)	0.002*	1.815(.875–3.764)	0.109
	>/ = 65	5.870 (2.479–13.900)	.000*	4.040(1.396–11.696)	.010*
Gender	**Male #**	1			
	Female	.832(.497–1.392)	0.483		
ASA Status	**ASA I #**	1			
	ASA II	2.756(1.605–4.731)	0.027*		
	ASA III	5.307(1.25–23.244)	.000*		
Coexisting disease	Yes	3.852 (2.157–6.881)	.000*	1.470(.597–3.625)	.402
	**No #**	1			
History of substance use	Yes	6.670 (3.374–11.914)	.000*	6.024 (3.173–11.435)	0.000*
	**No #**	1			
Pre-operative premedication with benzodiazepine	Yes	2.838 (1.230–6.547)	0.0014*	4.284(1.557–11.788)	0.005*
	**No #**	1			
Pre-operative anxiety	Yes	1.466(.785–2.789)	0.230		
	**No #**	1			

*. = Statistically significant.

#.and category written in bold are References.

Induction of anesthesia with ketamine was a risk factor for postoperative delirium with AOR of P-value of 0.047 as shown in Table 4 below.

**Table 4:** Anesthesia and surgery-related risk for postoperative delirium.

Variable		COR (95% CI)	P-value	AOR (95% CI)	P-value
Surgery type	**General surgery #**	1			
	Urology	1.409(.571–3.475)	0.456		
	Neurology	.618(126–3.419)	0.618		
	Cardiothoracic	.986(.087–11.237)	0.991		
	Gynecology	.798(.411–1.549)	0.504		
	Orthopedics	1.103(.555–2.191)	0.781		
Anesthesia type	**GA with ETT #**	1			
	GA with LMA	0.561(.114–2.762)	0.655		
	Monitored Anesthesia Care	2.943(.482–17.976)	0.242		
	TIVA	.589(.157–2.200)	0.431		
Induction agent	**Thiopental #**	1			
	Propofol	1.059(.533–2.103)	0.870		
	Ketamine	3.176(1.073–9.404)	0.37*	3.101 (1.02–10.567)	0.047*
	Ketofol	.847(.401–1.789)	0.663		
Postoperative analgesia given	Yes	1.058(.597–1.875)	0.846		
	**No #**	1			
Tracheal tube in situ	Yes	3.071(.504–18.723)	0.224		
	**No #**	1			
Urinary catheter in situ	Yes	1.650(0.923–2.950)	0.0.91		
	**No #**				

*. = Statistically significant.

#.and category written in bold are References.

NS= Not Significant.

## 5 Discussion

The emergence of delirium post-surgical procedures can be affected by different pharmacological and physiological factors. Therefore, management of emergence delirium was considered after investigating physiological and pharmacological factors. Intraoperative complications like lack of oxygen and accumulation of carbon dioxide are causes of hyperactive delirium postoperative period. other physiological conditions like low body temperature, low plasma glucose level, major electrolyte derangement, infection, different types of embolism, and fluid overload are highly related to the emergence of postoperative emergence delirium [4].

Numerous predictors have been documented for the development of postoperative emergence delirium. Geriatrics, current substance abuse, Coexisting disease, premedication with Benzodiazepine, induction with Ketamine, and ASA physical status were associated risk factor for postoperative emergence delirium depending on our results.

Age >65 years was 4 (p = 0.01) time risk factor for POED when we compare with age 18–40 years, our result was comparable with Pinho et al. study which compare age 69 years with 57 years with a p-value of 0.001 [12].

Preoperative use of benzodiazepines was a risk for POED AOR 2.8 (p = 0.014), this result was in line with a study with (p = 0.021) with benzodiazepines premedication and other surgical procedures like breast surgical procedure (P = 0.013), surgical procedure on abdomen (p = 0.014)and prolong surgical procedure (p = 0.001) risk factor for postoperative emergence delirium [13].

Tesfa et al. Study shows that old age above 60 years was (AOR = 7.8, 95% CI: 3.1, 19.5), and premedication with benzodiazepine (AOR = 11.3, 95% CI: 4.9, 25.8) were a risk factor for delirium which was in line with our study [14].

Regarding substances (alcohol, chat, and smoking) use of individuals around 6 times (p = 0.000) risk factor for POED in our study, so this study had a similarity with a study done by Assefa Solomon et al. studies show that the use of the substance by a community with (P = 0.001) indicated that the most powerful preoperative predictors of delirium were substance abuse. In our case, those patients showed aggressive behavior like catheter removal, IV line removal, and endotracheal removal immediate postoperative period in our PACU [15].

Another study shows that chronic smoker patients had a high risk for postoperative delirium with a p-value of 0.001. This study was in line with our findings since we investigate substance abuse components smoker, alcohol, and chewing chat [16]. Another study by Kim. et al. also state that high alcohol consumption had independent risk factor development of postoperative emergence delirium which was comparable with our finding [17].

Underlying comorbidity was a risk factor with CRO 3time more risk than patients without coexisting disease in our finding, which was comparable with TL.Janssen. eta al. study shows that renal impairment (OR 2.2), cognitive impairment (OR 4.1), ASA physical status ≥3 (OR 2), chronic smoker (OR 2.7), admission of intensive care unit (OR 7.1), blood transfusion (OR 2.4) and patients with a diagnosis of abdominal malignancy (OR 4.0) associated risk factor for postoperative emergence delirium [18].

The use of ketamine as an induction agent was a 3-time risk for POD in our study, which is comparable with punita t. et al. studies [19].

## 6 Conclusion

We conclude that older age, current substance use, Coexisting disease, Benzodiazepine exposures, Ketamine, ASA physical status, and coexisting disease were determinant associated risks for POED in a patient undergoing general anesthesia.

## Ethical approval

Ethical review board of Dilla University, College of medicine and health science issued the ethical clearance to do this research.

## Sources of funding

Dilla University has funded the research project.

The sponsor has no any role in the research activity.

The sponsor did not took part in any action of the research project other than funding.

## Author contributions

Teshome Regasa and Zemedu Aweke contributed to study conception, collected data, prepared manuscript, and performed statistical analysis. Derartu Neme contributed in statistical analysis. All the authors read the manuscript and approved the final submission.

## Registration of research studies

The research were registered on research registry platform with a unique identification number of researchregistry7363.

## Guarantor

Teshome Regasa.

## Consent

Verbal and written informed consent obtained from respondents before actual data collection. Confidentiality were maintained by removing any identifiers from the questionnaires. The participant were informed their right to participate and leave the study at any time without any risk.

## Provenance and peer review

Not commissioned, externally peer-reviewed.

## Declaration of competing interest

There is no conflict of interest to declare.

## List of Abbreviations

ASA: American Society of Anesthesiologist
ICU: Incentive care unit
POED: Post-operative emergence delirium
SBP: Systolic Blood Pressure
RASS: Richmond agitation and sedation scale
